# The second wave of the Controlled Antenatal Thyroid Screening (CATS II) study: the cognitive assessment protocol

**DOI:** 10.1186/1472-6823-14-95

**Published:** 2014-12-12

**Authors:** Charlotte Hales, Sue Channon, Peter N Taylor, Mohd S Draman, Ilaria Muller, John Lazarus, Ruth Paradice, Aled Rees, Dionne Shillabeer, John W Gregory, Colin M Dayan, Marian Ludgate

**Affiliations:** Thyroid Research Group, Institute of Molecular and Experimental Medicine, School of Medicine, Cardiff University, Cardiff, CF14 4XN UK; Department for Paediatric Psychology, St. David’s Children’s Centre, Cardiff, UK

**Keywords:** Hypothyroidism, Pregnancy, Child, Intelligence, IQ, Cognition, Motor coordination, Long term memory, Thyroid function

## Abstract

**Background:**

Children whose mothers had low thyroid hormone levels during pregnancy have been reported to have decreased cognitive function. The reported research is part of the follow-on study of the Controlled Antenatal Thyroid Screening Study (CATS I), a randomised controlled trial which investigated the impact of treated vs. untreated low thyroid hormone level in women during pregnancy with the primary outcome being the child’s IQ at age 3. No significant differences in IQ were found between the treated and untreated groups. These children are now aged between 7 and 10 years and aspects of their cognitive functioning including their IQ are being reassessed as part of CATS II.

**Methods/Design:**

Cognitive assessments generate an IQ score and further tests administered will investigate long term memory function and motor coordination. The aim is to complete the assessments with 40% of the children born to mothers either in the treated or untreated low thyroid hormone groups (n = 120 per group). Also children born to mothers who had normal thyroid functioning during CATS I are being assessed for the first time (n = 240) to provide a comparison. Assessments are conducted either in the research facility or the participant’s home.

**Discussion:**

The study is designed to assess the cognitive functioning of children born to mothers with low thyroid hormone levels and normal thyroid functioning during pregnancy. This is the largest study of its type and also is distinguishable in its longitudinal design. The research has the potential to have a significant impact on public health policy in the UK; universal screening of thyroid hormone levels in pregnancy may be the recommendation.

## Background

Subclinical Hypothyroidism (SH), defined as an elevated level of thyroid stimulating hormone (TSH) with normal circulating levels of free thyroxine (T4) and free tri-iodothyronine (T3) [1], affects 3-6% of the UK population [2, 3]. SH in pregnancy is defined as a TSH concentration higher than the upper limit of the pregnancy related reference-range with normal T4 (and, if measured, normal T3). The upper limit of TSH is now defined as 2.5 mIU/l in the first trimester and 3 mIU/l in the second and third trimesters [4, 5]. SH is a biochemical diagnosis as symptoms may be mild, non-specific and mimic typical symptoms occurring in pregnancy [6]. The recent lowering of the pregnancy TSH threshold has had a dramatic effect on the prevalence of Gestational SH (GSH) with prevalence rates in Belgium increasing from 2-3% to 6.8% [7] and over 15% in a recent large pregnancy screening study in the USA [8].

Iodine is an essential component of thyroid hormones and the UK population is borderline iodine deficient [9]. When iodine supplies are severely inadequate, TSH increases as a compensation mechanism. In women with chronic iodine deficiency during pregnancy, their depleted iodine stores are not able to compensate for increased iodine demands leading to increased risk of maternal goitre and hypothyroidism [10].

There is some evidence that neuropsychological and intellectual development of offspring can be adversely affected by an iodine deficiency during pregnancy [11–13] or GSH [14–18]. Intellect, as measured by Intelligence Quotient (IQ) tests has been shown to predict a range of life outcomes such as academic performance, job performance, years in education and even physical health [19–25].

The suggested mechanism for these effects of iodine deficiency and GSH is that although the brain is very dependent on thyroid hormones for normal development, active secretion of thyroid hormone in the fetus does not start until about 18-20 weeks gestation so the fetus is dependent on the mothers’ circulating hormones for growth and development up until this point [26]. The number of studies investigating the impact of an untreated GSH on an offspring’s IQ is growing but the findings are equivocal: in one retrospective study untreated GSH was shown to lower an offspring’s IQ by a mean of 7 points [14], and out of the 48 GSH offspring compared to 124 matched control children, 19% of these had an IQ of <85 compared to only 5% of the controls (IQ scores as measured on standardised tests are normally distributed so an average IQ score falls in the range of 90-109; with a percentile ranking of 50 for an IQ of 100).

Further studies have also shown a deficit of GSH having an impact on the offspring’s intelligence [14, 27–29]. Li [15] detected this impact on children as young as 25-30 months (compared to controls, mean intelligence scores were found to be significantly lower p = 0.008). There is also some evidence of more specific impairments to the offspring; motor coordination, attention, language and visuo-motor performance [14, 15, 29–31]. However other research, including prospective studies, have not found that GSH affects any aspect of cognition for the offspring [17, 32, 33].

If there is a significant impact of GSH on the child’s development, this could potentially be widespread and to test and treat for GSH is reasonably “low-cost” [34]. In response to these findings of an effect there are those who propose screening during pregnancy to help determine the circulating thyroid hormone levels in the mothers [34, 35]. However before that decision can be made the evidence needs to be more robust based on longitudinal large-scale randomised trials including women with treated and untreated GSH and their off-spring. The Controlled Antenatal Thyroid Screening (CATS) study is one such trial, understood to be the largest randomised controlled trial (RCT) of this kind to date.

Between 2002 and 2006 the CATS I study [36] recruited participants in the UK (10 centres) and Italy (1 centre: Turin). A total of 16,349 pregnant women with no known thyroid disease participated in the UK at a gestation of 16 weeks or less and a blood sample was obtained to measure TSH and T4 (A further 5,497 were recruited in Turin and included in CATS I but are not being included in this follow-on study; CATS II). TSH levels above the 97.5^th^ percentile, free T4 levels below the 2.5^th^ percentile, or both were considered as a Suboptimal Gestational Thyroid Function (SGTF) result. Women with SGTF in the screen group were treated during their pregnancy and those in the control group with SGTF were untreated (see article Lazarus [36] for further details). IQ of the offspring was measured at a mean age of 3.2 years with no significant differences found between the groups.

Whilst measuring IQ at age 3 has value as a general indicator of cognitive function, it does not give a detailed cognitive profile. It is not best suited as a longer term estimation of cognition function [37] which can be achieved by administering a more in-depth battery of assessments with older children. Therefore the primary aim of CATS II is to measure the children’s cognitive function at age 7–10 years. In addition to the cognitive assessments the study has expanded to include investigations of (i) the child and mothers’ bone mineral density (ii) height, waist and weight measurements, blood pressure and arterial stiffness (iii) DNA collection (iv) and behavioural questionnaires. The outline of these additional investigations will be included but the details are outside the range of this protocol which focuses on the primary outcome; the child's cognitive functioning.

## Methods

CATS II is a follow-on study from CATS I, a large multi-centre RCT, that aims to investigate the possible long term effects of exposure to SGTF. Cognitive assessments are administered to children aged between 7 and 10 years to ascertain their overall development. The study is aiming to recruit a total of 480 participants between August 2011 and the end of August 2015. Participants can be seen in either the research centre or can choose to be visited in their homes. If the participants prefer they can opt to provide a reduced data set using a post pack. The study was approved by the Wales Research Ethics Committee 2 and Caldicott Guardian.

### Eligibility

Participants are included if they were involved in CATS I and originally recruited from the UK, and are approached for CATS II when the child involved in the study is ≥7 years 0 months to ≤ 10 years 11 months. Participants are excluded from the study if they have moved overseas.

### Sample size justification

With a 5% two-sided significance level and 90% power, a sample of 120 from both treated and untreated SGTF groups will allow a detection of a difference of 6 IQ points in mean IQ (assuming mean IQ to be 100 with a standard deviation (SD) of 15 points) [38].

We have used an increase in the odds of IQ < 85 as a primary outcome in treated and untreated SGTF groups, similar to the CATS I analysis. We have 80% power with a 5% two-sided significance level to detect a 1.97 increase in odds of IQ < 85 in untreated SGTF offspring assuming treated SGTF offspring have a mean IQ of 100 with a SD of 15. This is substantially lower odds than that observed comparing low thyroid hormone bioavailability to those offspring of mothers with normal GTF bioavailability (odds ratio = 2.36). In addition 240 participants (1.5%) from the normal GTF group who are randomly selected from the complete group of 15,744 from the UK cohort will be used to assess whether there is interaction with maternal thyroid status and levothyroxine treatment on offspring IQ.

### Recruitment

**Figure 1:**
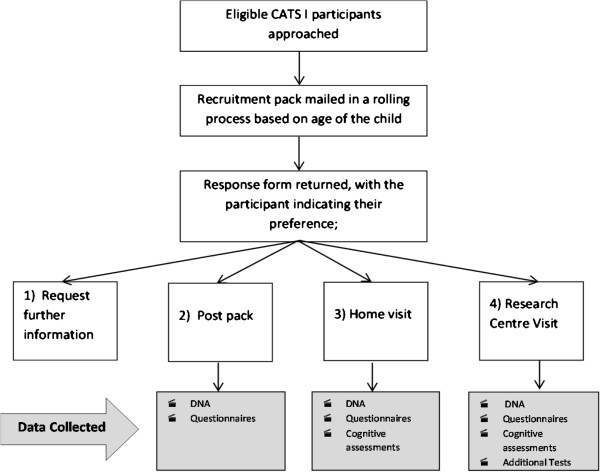
**CATS II recruitment process.**

An initial contact pack inviting the CATS I mothers to participate in the research is mailed to all in the SGTF groups. Re-involvement in to CATS is centred on the age of the child; those born earliest during CATS I are contacted first in a rolling recruitment process over the 48 months of the project. The Welsh Demographics Service and Patient Data Registrar have been utilised to ensure up-to-date addresses for individuals from the SGTF groups. Figure 1 details the recruitment process for participants.

Those participants, who wish to complete the cognitive assessments at their home or by attending the research centre, are contacted to arrange the appointment and post packs are mailed out. Research centre appointments take 2.5 hours, home appointments last for 2 hours or less.

A different letter is sent to individuals from the normal GTF group who consented to participate in CATS I but did not participate in the cognitive assessments. There are 15,744 potential participants that could be contacted from the Wales CATS I cohort for re-involvement into CATS II. 240 participants are required from this group for CATS II. As participants are contacted by year of registration into the study a random selection is mailed from each of the 4 years CATS I recruited for, totalling 5,000 packs being sent to mothers from the normal GTF group.

### Cognitive measures and procedure

To be able to address the primary aim of CATS II, an IQ test (1) and additional cognitive tasks (2) are administered to the children in the study.

1. The Wechsler Intelligence Scale for Children- Fourth Edition UK (WISC). The WISC [38] provides sub-test and composite scores that represent intellectual functioning across specific cognitive domains: Verbal Comprehension (VC); Perceptual Reasoning (PR); Working Memory (WM); and Processing Speed (PS). The IQs generated from these areas equally contribute to the Full Scale (FS) IQ.
2. Developmental NEuroPSYchological Assessment- Second Edition (NEPSY). Possible delays in long term memory (LTM), WM and fine-motor skills are investigated by sub-tests from the NEPSY [39]: List Memory and List Memory Delayed (combined score) (LTM), Memory for Designs (WM), Memory for Designs Delayed (LTM), Fingertip Tapping (motor) and Narrative Memory (WM).

If the participants opt to attend the research centre, the child is assessed in a clinical environment. If participants select to be seen at their homes, a table and a quiet room with minimal distractions are requested. The tests are administered in a set order with standardised verbal instructions given to each child. The WISC is administered first, followed by the NEPSY.

The WISC raw scores are calculated by hand; an automated WISC scorer software is then used to calculate the scaled and composite scores and generate a report for the parents. This details the composite scores, percentile ranks and classifications (e.g. average, high average etc.) of the FSIQ, VCIQ, PRIQ, WMIQ and the PSIQ. If the participants have any questions regarding their child’s results, they are able to directly contact the examiner (a psychologist) who completed the assessments.

There is only one examiner on the project to increase reliability. Training was completed for cognitive test administration. A randomly selected 10% of the completed assessments are double scored to ensure reliability. Means are reviewed fortnightly to ensure no scaled or composite scores are well above or below the average which may indicate skewed testing. There is also an ongoing evaluation of age and gender distribution between normal GTF and the SGTF groups.

### Statistical analysis

The main statistical analysis will be executed according to the CATS II analysis plan. The data will be analysed using STATA version 12. Dataset accuracy will be scrutinised using histograms and cross tabulations to identify any outliers and errors in the data set. Implausible values (>4 SD from the mean) will be considered as outliers and will be recoded as ‘missing’. Descriptive statistics will be presented as means, SD, medians and lower and upper quartiles. All variables will be standardized; analyses will therefore be presented as per SD, for linear regression analysis. Odds ratios will be assessed for FSIQ thresholds.

Primarily, a univariable analysis will be commenced followed by subsequent multivariate analyses to adjust for key potential confounders relating to schooling and social class. Logistic regression will be used to identify if untreated SGTF offspring have increased odds of an FSIQ < 85 compared to treated SGTF offspring. Children of normal GTF mothers will be included in a secondary analysis to assess the odds of an FSIQ <85 by maternal thyroid status; with an interaction term for treatment status to enable us to see if treatment reduces the risk of low FSIQ.

The following four models of analysis will be used to explore the data;

- Model 1; Crude
- Model 2; adjusted for child sex
- Model 3; adjusted for model 2 and age of mothers at birth of offspring and whether the child was breastfed.
- Model 4; adjusted for model 3 and schooling (Welsh or English school attended) and socioeconomic background.

The primary analysis for the study is assessing the odds of FSIQ below 85 in children of mothers who had SGTF compared to children of mothers with normal GTF, calculated by a logistic regression. An interaction term will be included for treatment vs. no treatment. This outcome will therefore utilise our entire CATS II cohort and is similar to CATS I in that it also investigates FSIQ 1 SD from the mean.

Secondary analysis outcomes specific to the cognitive data collection will investigate multiple aspects. Firstly, we will explore whether children from the untreated SGTF groups have an increased odds of a VCIQ <85 and if their LTM and motor coordination is significantly lower than offspring from mothers with treated SGTF. Secondly, we will investigate if there is evidence of non-proportional odds of a lower FSIQ in children of treated compared to untreated SGTF mothers’ using likelihood ratio tests on generalized ordered logistic thresholds to assess if the data best fits a proportional or non-proportional model. Furthermore, by applying data from our offspring of normal GTF mothers, we can explore the odds of this group having a higher FSIQ than children of treated SGTF mothers; this will include a non-inferiority analysis. Likewise, by comparing the normal GTF to the untreated SGTF data, we can investigate whether maternal TSH predicts FSIQ. Additionally, we can investigate whether maternal TSH and poor DIO2 (a gene involved in activating T4 to T3) in the offspring predicts FSIQ; this gene will be genotyped in CATS’ children using standard protocols. This final secondary analysis will use data from the normal GTF and untreated SGTF groups and from this we could consider whether a treatment would reduce the risk of having lower FSIQ.

Final investigations of the cognitive data will take the form of a sensitivity analysis. CATS I and CATS II VCIQ, PRIQ and FSIQs can all be compared; which will assist identifying whether there is any bias found in CATS II in relation to those participants recruited to CATS I. The WISC utilises scaled scores to calculate IQs, and the NEPSY also reports scaled scores. Therefore comparisons between the WISC WM scores can be compared to the NESPY WM scores. Finally, an imperative sensitivity analysis will look at the extent to which offspring of mothers whom had normal GTF represent a normal population i.e. IQs at a mean of 100 and NEPSY scaled scores at a mean of 10 for each subtest.

## Discussion

The aim of this paper is to describe the study protocol of the cognitive aspects of the CATS II research project. By conducting this study, we hope to better understand the impact of maternal hypothyroidism and whether treatment for such should be sought. We will add to the wider literature for SGTF and contribute to knowledge in clinical, epidemiology and psychology fields. The current research is, to the authors’ knowledge, the largest of its kind in the world to have a treated compared to untreated model of SGTF at this age group; it has the potential to have a significant impact in the UK and may be policy changing; universal screening for SGTF may be the outcome based on the study recommendations. If treatment for SGTF is found to have an impact on FSIQ, it is our aim to revisit these children to repeat assessments at age ≥14 years. CATS II recruits when the children are aged between 7–10 years; one of these reasons being that IQ discrepancies of offspring of normal GTF and hypothyroid mothers during pregnancy, were identified in children aged 7 [14]. A further reason for assessing children at this age is related to our further investigations for CATS II; specifically the bone mineral density measurements, these should be taken before the confounding factors of puberty occur.

The key strength of this study is assessing the offspring at a second time point during their lives. We are not aware of any other studies in this field that have completed this. By being flexible in our outcome measurements for the participants, we are able to maximise recruitment. A further strength is the volume of data collected; bone density, DNA samples, child behavioural questionnaires are just some of the data the participants will provide. The main challenge of this study will be the recruitment targets and the rate of attrition. A biased sample in the normal GTF group may present itself; individuals better equipped to aid the study, for example with free time will more readily volunteer. This bias may only present in the normal GTF group, as those from the SGTF groups took part more recently (when the child was aged 3.2) and are more likely to remember their participation.

In summary, CATS II is a follow-on study from a large RCT collecting a wealth of data with the primary aim of investigating FSIQ of offspring born to mothers who had SGTF compared to those with mothers who had normal GTF. Regardless of outcome, the results will have an effect of how an underactive thyroid during pregnancy is perceived, and more importantly, whether it is imperative to treat or not.

## Abbreviations

CATS: Controlled antenatal thyroid screening
SH: Subclinical hypothyroidism
TSH: Thyroid stimulating hormone
T4: Free thyroxine
T3: Free tri-iodothyronine
GSH: Gestational subclinical hypothyroidism
IQ: Intelligence quotient
RCT: Randomised controlled trial
SGTF: Suboptimal gestational thyroid function
SD: Standard deviation
WISC: Wechsler intelligence scale for children-fourth edition UK
VC: Verbal comprehension
PR: Perceptual reasoning
WM: Working memory
PS: Processing speed
FS: Full scale
NEPSY: Developmental neuropsychological assessment-second edition
LTM: Long term memory.
